# Acute Syphilitic Meningitis

**Published:** 1917-02

**Authors:** Boris Bronstein

**Affiliations:** Odessa, Russia


### Abstracts and Selections.

---

### Acute Syphilitic Meningitis.

---

BY BORIS BRONSTEIN, M. D., ODESSA, RUSSIA.

---

Bronstein considers that the term acute syphilitic meningitis should be more particularly applied to acute meningeal phenomena of the secondary period, sometimes preceding, but more frequently accompanying the cutaneous manifestations of this period. The pathology is essentially a meningovascularitis with hypersecretion of the cerebro-spinal fluid. Prodromal symptoms, such as headache and insomnia, may or may not occur. Acute syphilitic meningitis at its height, as Bronstein says in the December *International Clinics,* presents the clinical picture of the tubercular form, differing from the latter by the indistinctness of the symptoms, such as contractures and stiffness of the neck, and by the absence of any marked disturbance of the pulse and respiration. In the luetic form fever is apt to be present and there may be remissions and relapses. Lumbar puncture reveals a considerable hypertension of the cerebro-spinal fluid, albumin in quantity, and a marked lymphocytosis with plasmozellen. The cerebro-spinal fluid may yield a positive Wassermann even when the blood serum is negative. Other manifestations of syphilis are to be looked for. The immediate prognosis is rarely fatal but the ultimate prognosis should be reserved. Prophylactic treatment is recommended whenever the cerebro-spinal fluid shows a lymphocytosis, even when all meningeal symptoms are wanting. The treatment consists in frequently repeated removal of the cerebro-spinal fluid in considerable amount, combined with intravenous injection of cyanide of mercury and intraspinal injections of colloidal mercury. Neosalvarsan or salvarsan have a much more rapid action, but must be prudently handled in neurologic lesions of syphilis.—*International Clinics.*

